# Left and Right Ventricle Leads Switch as a Solution for T Wave Oversensing - How a Good Idea Turned Out Bad

**DOI:** 10.1016/s0972-6292(16)30757-4

**Published:** 2014-05-25

**Authors:** BSN Alzand, TJE Phlips, R Willems

**Affiliations:** 1 Department of Cardiology, Glorieux General Hospital, Ronse, Belgium; 2 Department of Cardiology, OLV General Hospital, Aalst, Belgium; 3 Department of Cardiology, University Hospitals Leuven, Leuven, Belgium; 4 Department of Cardiovascular Sciences, University of Leuven, Leuven, Belgium

**Keywords:** T-wave ovesensing, CRT-D, inappropriate socks

## Abstract

A 50-year-old male with a CRT defibrillator received inappropriate ICD shocks due to T-wave oversensing. Decreasing the sensitivity to avoid T wave oversensing was not an option due to a suboptimal R-wave sensing amplitude. We decided to re-plug the LV lead in the RV port and the RV lead in the LV port. This however led to intermittent phrenic nerve stimulation due to mandatory bipolar (tip-ring) or unipolar (tip-can) pacing on the LV-lead from the RV port. Re-intervention was necessary with the implantation of an additional pacing/sensing RV lead. A software programmable choice to switch sensing and tachycardia detection from RV to LV lead could be a valuable feature in future CRT devices.

## Case report

**Figure 1:**
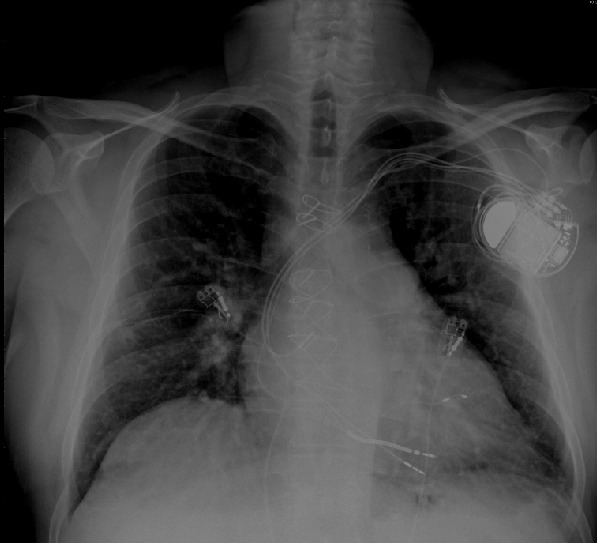
Chest X-ray, postero-anterior view showing a BiotroniK CRT-D with a RA lead, RV shock lead, and LV lead in the coronary sinus. Note the wire sutures post-sternotomy due to previous coronary artery bypass grafting.

**Figure 2:**
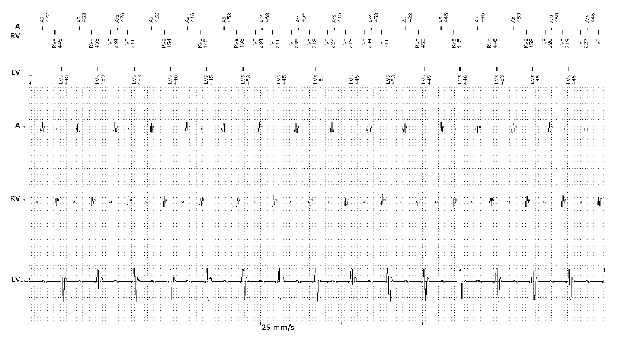
EGMs form the Biotronik Lumax 540-HF-T, showing inappropriate detection of ventricular fibrillation due to T-wave oversensing. No T-wave oversensing is noted on the LV lead.

**Figure 3:**
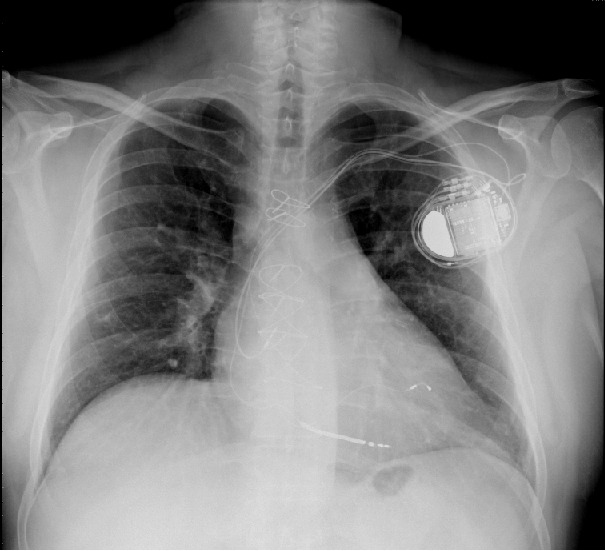
Chest X-ray, postero-anterior view showing a BiotroniK CRT-D with a RA lead, RV high voltage lead, RV pacing/sensing lead and LV lead in the coronary sinus.

We present here a case of a 50-year-old male known with multiple coronary stents, coronary artery bypass grafting, ischemic cardiomyopathy, low ejection fraction of 28%, paroxysmal atrial fibrillation, left bundle branch block and NYHA class III. The patient was implanted with a CRT-D (Lumax 540-HF-T, Biotronik) with a with a single coil high voltage lead (BiotronicLinox S65), active fixation bipolar atrial lead (BiotronikSiello S53) and bipolar left ventricular lead (BiotronikSiello S53) 2 years ago (Figure 1). He was on beta-blockers, ACE-inhibiters, oral wafarin, statins, amiodarone and aldosterone receptor antagonist. He was urgently referred to our hospital due to recurrent ICD - shocks during exertion. Interrogation of his ICD showed a normal RA, RV, LV and shock lead impedance of 483, 701, 456 and 61 Ohm respectively. RA, RV and LV sensing of 3.9, 1.5 and 20 mV, respectively. The RA, RV and LV pacing thresholds were 1 V@ 0.4 ms, 0.8 V @ 0.4 ms and 0.6 V @0.4 ms (LV ring - RV coil), respectively. Analyzing the electrogams (EGMs) revealed inappropriate ICD shocks due to T-wave oversensing (Figure 2). We decided to perform an invasive procedure to re-plug the LV lead in the RV port and the RV pacing/sensing lead in the LV port. In the catheterization-lab we reopened the ICD-pocked, switched the leads successfully and performed a defibrillation threshold test without any technical hitches. Patient was discharged free of complaints. However, few weeks later he contacted us because of intermittent diaphragm stimulation in left sided decubitus position. These complaints were intolerable for hem and a re-intervention was necessary. An new procedure was performed, the LV lead was repluged in the LV port and a new RV pacing/sensing lead was implanted and connected to the RV pacing/sensing port (Figure 3). Patient remained free of complaints during follow-up.

## Discussion

As a tertiary center, we were confronted with a patient suffering form repetitive inappropriate ICD-shocks due to T-wave oversensing. Despite the enhanced T wave suppression algorithm and the programmed RV sensitivity of 0.9 mv, T-wave oversensing remained. Increasing the programmed sensitivity > 0.9 mv to avoid T wave oversensing was deemed inappropriate because it might lead VF/VT undersensing in view of the low R-wave amplitude of 1.5 mV. An invasive procedure was inevitable. One of the solutions for this problem is to reposition the existing RV lead or to implant a new pacing sensing lead. We came up with a less invasive solution and decided to re-plug the LV lead in the RV port and the RV pacing/sensing lead in the LV port. Comparative cases using LV sensing has been describe in the literature to overcome sensing problems in arrhythmogenic right ventricular dysplasia [1,2] Sensing on the LV lead in our patient was superior, i.e. 20 mV and T wave sensing was not present. By switching the leads, sensing and tachycardia detection will be conducted form the LV lead (RV port). This option seemed less invasive than the other alternative options like repositioning the existing RV lead or implanting a new RV pacing/sensing lead. Important pitfalls are the LV lead stability [1] and pacing polarity. The LV lead stability is not an issue in our case as it has been implanted for more than 2 years. The pacing polarity was vital as it has been programmed Unipolar LV ring - RV coil. The reason of using this configuration was due to diaphragm stimulation at higher outputs with bipolar LV tip - LV ring and unipolar tip - RV coil configurations during the initial implantation procedure. Bipolar pacing/sensing configuration is mandatory in the RV port. We have tested bipolar LV pacing and found an acceptable margin between the bipolar LV pacing threshold and the diaphragm stimulation threshold and decided to proceed with the LV-RV leads switch. Few weeks later, patient came back with intermittent diaphragm stimulation in left sided decubitus position. A second procedure was performed and a new pacing/sensing lead was implanted. A software programmable choice to switch sensing and tachycardia detection from RV to LV lead could be a valuable feature in future CRT devices. Such an option would have been appropriate in this case and spared our patient an invasive procedure.

## References

[R1] Bilchick KC (2006). Use of a coronary sinus lead and biventricular ICD to correct a sensing abnormality in a patient with arrhythmogenic right ventricular dysplasia/cardiomyopathy. J CardiovascElectrophysiol.

[R2] Lochy S (2010). Left ventricular sensing and pacing for sensing difficulties in internal cardioverter defibrillator therapy for arrhythmogenic right ventricular cardiomyopathy. Europace.

